# Rutgers Youth Enjoy Science Program: Reducing Cancer Health Disparities by Reducing Education Inequities

**DOI:** 10.15695/jstem/v5i2.09

**Published:** 2022-08-31

**Authors:** Sunita Chaudhary

**Affiliations:** Research Education, Rutgers Cancer Institute of New Jersey, New Brunswick, NJ

**Keywords:** Underrepresented Minority, Research Career, Science Education, Health Disparities

## Abstract

The goal of the Rutgers Youth Enjoy Science Program (RUYES) is to increase the diversity of the cancer research workforce. RUYES provides hands-on mentored cancer research experience and curriculum development support to high school science teachers. RUYES also engages high school and undergraduate students from underrepresented backgrounds (URG) in mentored cancer research and professional career development activities. Rutgers Cancer Institute faculty members with active, well-funded research programs and extensive mentoring experience serve as research mentors. In addition, RUYES provides support to participants to develop innovative cancer related outreach activities to connect with families and communities of participants. Teachers and students engage in research and program related activities for a total of 12 weeks per year, for two years. Teachers engage in cancer research for eight weeks each for two consecutive summers (sixteen weeks total). Collaborative partnership with Rutgers Office of STEM Educations supports teachers in developing novel instructional approaches that relate to their research experience. Students have the opportunity to conduct cancer research for ten weeks each for two consecutive summers (twenty weeks total). Students receive co-curricular and professional development support throughout their participation. In addition, teachers and students engage in post-summer program engagement for 4 and 2 weeks, respectively. We recruit program participants from all over New Jersey with special emphasis on school districts with high percentages of students from URG. This report details the components of the RUYES program, outcome evaluation plan, successes and challenges, and lessons learned for others interested in establishing similar programs at their institutions.

## INTRODUCTION

Cancer incidence rates are estimated to rise from 1.6 million in 2010 to 29.5 million by 2040 (NCI, 2020). Individuals from underrepresented minority populations bear a disproportionate burden of cancer with higher incidence and mortality rates (Kamangar, 2006; O’Keefe, 2015; Smith et al., 2009). Factors contributing to disparities in cancer care access and outcome are complex. Increasing diversity of the oncology workforce is an effective strategy to increase cancer health equity. A diverse workforce improves access to cancer care and improves cancer outcomes for disparate populations by encouraging policymakers to make effective recommendations, which address the needs of a diverse population (Winkfield, 2017).

### Needs Assessment and Goals.

With 21 counties, New Jersey (NJ) is one of the most racially, ethnically and socioeconomically diverse regions in the US. The State is home to a large immigrant and minority population and disparities in education level, English language proficiency, immigrant status, income, population density, and access to cancer care. The three major cities in the State - Newark, Trenton, and Camden - have a large population of minority, low income, and immigrant residents. New Jersey has significant cancer health disparities and poor outcomes in members of culturally and racially underserved communities (Kulkarni, 2019; Yang, 2020, NJDOH Data Briefs, 2015–2019).

Research studies show that lack of diversity in the oncology workforce contributes to inequalities in cancer care and outcomes (Winkfield, 2017; Hamel et al., 2015). Increasing workforce diversity stems cancer health inequities and improves outcomes in minority cancer patients by influencing research priorities and cultural competence of health care providers (AACR Cancer Disparities Progress Report 2020). However, barriers preventing students from underrepresented groups (URG) from entering the oncology workforce remain (Winkfield, 2017). Demographic data collected by American Society of Clinical Oncology (ASCO), found that compared to several internal medicine subspecialty fellow-ships the proportions of black/African American and Hispanic/Latino oncology fellows were lower in the oncology workforce (Haynes and Smedly, 1999). To address the lack of diversity, ASCO recommended establishing a longitudinal pathway for increasing diversity (Winkfield, 2017).

Research focusing on underrepresentation of minorities in the broader biomedical workforce supports educational pipeline programs for students early on during their academic training, for increasing diversity in health professions (Smith et al., 2009; Barlow and Villarejo, 2004). Furthermore, pipeline interventional programs that include research experiences, have a positive impact on several key attributes for entry and retention of students from underrepresented groups (URG) in biomedical fields (Hunter et al., 2007; Hurtado et al., 2009; Silverstein et al., 2009).

Studies show that teacher involvement in scientific research also benefits students (Silverstein et al., 2009). Students of high school science teachers who engaged in summer research experience had improved scores in standardized tests after their teachers participated in research (Silverstein et al., 2009). High school science teachers participating in mentored research experiences report improvements in science content knowledge, perceptions and confidence levels in process and content that influence their own science teaching and translatability to their students (Cutucache et al., 2017). Furthermore, such experiences inculcate positive networks and collaborations between researchers, teachers, and schools.

The overall goal of the Rutgers Youth Enjoy Science (RUYES) Program at Rutgers Cancer Institute is to attract and train students from URG (early on in their academic trajectory) in cancer research and healthcare careers. To achieve the goal RUYES provides a multipronged yet integrated experiential learning experience to high school science teachers and youth from URG. RUYES components include research and curriculum development activities for high school science teachers; hands-on research training and professional development activities for students from URG; and outreach to middle school students, their families and communities. Experienced leadership team with record of accomplishment in developing, implementing and evaluating pipeline programs for students from URG, supports the Program.

The Program takes advantage of synergistic collaborations that leverage resources and expertise of similar programs across Rutgers and other regional institutions. Both formative and summative evaluation by an external evaluator are integral parts of the Program. Rutgers Cancer Institute, the state’s only NCI designated Comprehensive Cancer Center provides a multi-disciplinary research environment, robust educational and outreach resources for RUYES.

### Background.

RUYES builds on and expands the scope of the highly successful Continuing Umbrella for Research Experience (CURE) pipeline program. Funded by the National Cancer Institute, Center to Reduce Cancer Health Disparities, CURE targeted highly motivated students from URG in the local New Brunswick community. CURE recruited sophomore or junior high school and undergraduate students from the New Brunswick Health Sciences Technology High School (NBHSTHS) and Rutgers, New Brunswick, respectively. Students engaged in mentored hands-on research, academic, and professional enrichment activities for full time during summer and half day per week during the academic year for two years. The first cohort of eight students (four high school and four undergraduate) recruited in 2004, completed training in 2006. From 2006 through 2019, the CURE program successfully trained 102 participants from under-represented groups in cancer research, contributing 8–9 high school and undergraduate students annually to the biomedical workforce pipeline. Summarized below are key outcomes of the CURE participants from 2004–2019.

- Forty-seven high school students successfully completed the program. All of these students (100%) pursued higher education and thirty-nine (83%) of them selected STEM majors in college.
- Eighteen CURE alumni who participated at the high school level are pursuing biomedical careers including pharmacy, nursing, occupational therapy, pre-med and PhD in Forensic Science.
- Of the fifty-seven undergraduates who successfully completed the program a vast majority (n=52; 91%) pursued graduate studies in STEM including PhD, MD, MD, PhD at top-tier programs including Harvard Medical School, University of Pennsylvania, and University of Southern California etc.
- Thirty-five of the undergraduate alumni of the CURE program are also now in the biomedical workforce as physicians, pharmacists, and researchers.

Formative and summative evaluation of the student participants and mentors in the CURE program, using biannual surveys and interviews, provided important feedback to help in designing the components of the current RUYES program. Key lessons learned included:

- Provide broad overview of cancer as a disease to facilitate integration of students in their research experience.
- Provide opportunities for students to interact with each other so that they cultivate a sense of belonging and feel a part of the community of learners that supports each other.
- Develop partnerships with school and families of the students so they feel supported and nurtured.
- Be cognizant of the barriers faced by the students and help them in overcoming them.

## RUYES PROGRAM DESIGN

With funding from the National Cancer Institute (NCI) in 2020, the Rutgers Youth Enjoy Science Program replaced the CURE program. RUYES Program includes the following new components for significantly broadened scope and impact.

1. Recruitment from across the entire state of New Jersey (our catchment area);
2. Recruitment of high school science teachers and students;
3. Curriculum development support to teachers to create novel cancer focused Problem Based Learning curriculum aligned with NGSS standards;
4. Professional development support to students;
5. Community outreach activities by RUYES trainees for middle school students, families, and communities.

### Theoretical Framework.

Theory of Planned Behavior and Social Cognitive Career Theory provide a structural framework for the RUYES program.

#### Theory of Planned Behavior (TPB).

The Theory of Planned Behavior accounts for the influence of perceived control on behavior in addition to individual attitudes and subjective norms (Fishbein, 1975). According to this theory, intention to perform the behavior determines a person’s actual behavior. An individual’s positive or negative feelings toward the behavior (motivation) influence intention. The subjective norms (expectation of other individuals important to the individual) about the performance of the behavior (family/peer influence); and the individual’s perception of the ease with which the behavior can be performed (beliefs about factors that may aid or impede performance of the behavior, self-efficacy) influence intention. TPB addresses the limitations of the theory of reasoned action (Ajzen, 1985; Ajzen, 1991).

#### Social Cognitive Career Theory.

Social Cognitive Career Theory (SCCT; Lent et al., 1994; Lent et al., 2000), builds on the social cognitive theory originally proposed by Bandura (Bandura, 1994) and interprets the word “career” as “interest and choice processes” of an individual in academics or career. The SCCT postulates that individual career decisions and therefore earlier choices of academic majors, are influenced by the three personal variables namely, self- efficacy beliefs, intent to pursue a research career, and goals. These beliefs/choices are continually evolving due to the influence of cognitive processes, environment, and contextual variables (supports and barriers) of an individual. Self-efficacy, an important construct of the SCCT theory is an individual’s belief in their capability to perform an activity and take action to achieve a designated level of performance (Bandura, 1994). Strong self-efficacy beliefs and positive expectations of outcomes in a specific career are related to inherent commitment, deep interest and subsequently goal achievement in that career. Weak self-efficacy beliefs are linked to low commitment, aspirations and a failure to achieve goals. In addition, person inputs (race, gender, socioeconomic status), proximal and distal contextual variables (environmental barriers, parental support) provide indirect but never-the-less critical influences on the career development of an individual (Lent et al., 2000). These perceived barriers and supports vary by individual and situation and impact access to learning experiences, which influence self-efficacy beliefs and outcomes expectation.

#### Common Themes Between TPB and SCCT.

Both theories share several common themes. First, the interaction of intrinsic person-related factors and extrinsic influences of the person’s environment influence career related behavior. Second, individual’s beliefs about their own self, play an important role in the decision-making process. Third, perceived barriers and supports in an individual’s environment, which includes institutional environment and “important others” (parents, mentors, teachers), influence the decision-making process continually. Fourth, ethnicity, gender and socio-economic status have a direct overarching influence on all factors. Last career choices (academic majors) are not static but in a dynamic flux. Elements from both the theory of planned behavior and social cognitive career theory provided a conceptual framework for RUYES program design.

### Participant Recruitment and Selection.

RUYES is open to teachers and students from New Jersey with consideration on five urban school districts including New Brunswick, Newark, Paterson, Trenton and Camden with high percentage of students from underrepresented backgrounds. While New Jersey is one of the most diverse states in the country, significant racial disparities exist among the various school districts. Urban areas including Newark, Paterson, Trenton, Camden and New Brunswick tend to enroll mostly African-American or Latino students while suburban schools have mostly white students (Orfield, 2017). New Jersey has 48 public universities that offer undergraduate degrees. Each of the five target districts listed above has a four-year college and university that ranks in the top 10 for diversity in NJ. RUYES recruits participants from all over NJ with special emphasis on colleges and universities in the target districts. Recruitment efforts include advertisement of the program through Program website, brochures and flyers, and networks of other collaborating Programs (details under Outreach).

### Participant Selection and Match with Research Mentor.

RUYES website, brochure, flyers and recruitment emails provide the RUYES eligibility criteria, list of Research Mentors and descriptions of their research projects. Interested applicants apply through an online portal on the RUYES website, after answering a short survey to determine their eligibility. Complete application material includes demographic, academic information, Personal Statement including goals, and letters of recommendation. An Applicant Selection Committee consisting of the Program Director, program coordinators and two faculty research mentors review the applications and select applicants for acceptance. Selection is based on the applicants’ interest in conducting research, developing novel instructional approaches to take back to their school (only for teachers), and letters of recommendation. A final list of applicants who met the selection criteria is developed and sent to research mentors who have agreed to accept RUYES trainees. The mentors select 2–3 candidates who fit their research interests and meet them to discuss mutual research interests and establish compatibility. The program coordinators subsequently work with the mentors to honor their selections and match the mentor’s choices with those of participants as closely as possible.

### Research Experience.

RUYES provides mentor supervised hands-on research training in basic, clinical or behavioral cancer research to high school science teachers; and high school and undergraduate students for 8 to 10 weeks respectively, in summer. During this time, participants fully engage in their research experience and work hands-on an actual research project as a member of a team on an existing project of research investigator (mentor) at Rutgers Cancer Institute. Participants have access to an exceptional group of investigators organized in multi-disciplinary research teams focusing on a broad spectrum of cancer research organized into five programmatic areas. These include: 1) Cancer Metabolism and Immunology (CMI); 2) Clinical Investigations and Precision Therapeutics (CIPT); 3) Genomic Instability and Cancer Genetics (GICG); 4) Cancer Pharmacology; and 5) Cancer Prevention and Control. They interact with a diverse group of undergraduate, graduate, postdoctoral and faculty researchers on projects related to their academic and career interests. Projects include (not limited to): Tumor Biology, Bioinformatics, Psychosocial/Behavioral aspects in oncology, Pharmacy and Population-based settings. Some examples of research projects of participants during summer of 2021 include: Qualitative Analysis of Lung Cancer Screening Implementation in New Jersey; Role of LIF (Leukemia Inhibitory Factor) in Cancer Cachexia; Immune Checkpoint Protein Expression in Pancreatic Ductal Adenocarcinoma (PDAC); Correlates and Predictors of Childhood Sun Safety: A Systematic Literature Review; Robert Wood Johnson Barnabas Health System healthcare professional survey regarding lung cancer screening and tobacco treatment practices; How Framing Impacts Social Media Engagement in Health Communication.

### Mentoring.

Strong mentors are a critical part of the meaningful learning experience of the participants. Many studies have examined the role of mentoring in academic settings (Villarejo et al., 2008; Davidson and Foster-Johnson, 2001). Under-represented minorities and women have historically had limited access to traditional mentoring relationships (Brown et al., 2021). In our program, in addition to matching the participants to a laboratory, two peer-mentors are also assigned to them. The faculty research mentor assigns a senior graduate student or a post-doctoral student as a research peer-mentor to bridge the gap between the student and the principal investigator and help in student adjustment and learning. Research peer-mentors assist the student in the formulation of the research project; provide guidance in laboratory techniques and analysis methods, and in the participant’s research project. A second career peer-mentor assigned by the Program Director is either a student from underserved background selected from the participants of one of the diversity focused undergraduate or graduate programs at Rutgers or a RUYES alum. The role of the second career peer-mentor is to serve as a role model, encourage, share information about college preparation, college survival skills, study strategies, and advice on career development etc. This pairing is expected to foster academic and social support systems for the students, and promote a sense of belonging to the scientific community. Post-doctoral fellows and graduate students serving as mentors in the program participate in a *Culturally Aware Mentor Training Workshop*. The workshop helps mentors to identify their personal assumptions, biases, and privileges that may operate in their mentoring relationships. Through group discussion, case studies, and role-play, mentors have the opportunity to learn and practice culturally aware mentoring skills. The National Research Mentoring Network, which has a chapter at Rutgers, New Brunswick offers this workshop. The Center for Graduate Recruitment, Retention, and Diversity, at Rutgers University facilitates this workshop.

### Curriculum Development Support.

Teachers participating in the program use their research experience to develop effective science instructional and learning strategies to take back to their students in their schools. Development of novel curriculum approaches is facilitated through extensive support provided by experts in cancer focused curriculum and professional development. Each year (during two years of program participation), teachers meet with expert facilitators during weekly sessions. During these sessions, they discuss key materials for creating curriculum components. Two half-day workshops in the last two weeks of summer are organized for teachers to create curricular/instructional units that align with their goals and context of their local environment. This component of the program leverages partnerships with two Rutgers departments, the Center for Mathematics, Science and Computer Education (CMSCE) and the School of Public Health (SPH). Both these departments at Rutgers bring complementary expertise. The CMSCE conducts professional development programs in the STEM field for K-12 teachers, at the Center and in-district. These professional development programs are aligned with Next Generation Science Standards. While CMSCE has expertise in developing profession development programs for teachers in the broader STEM field, SPH brings strengths in developing cancer-focused curriculum targeted to high school students.

### Professional Development Support.

Providing a meaningful learning experience to participants at high school and undergraduate level is crucial for the success of research training programs. If specific efforts are not undertaken there is a risk of participants becoming mere “lab-hands.” To address this need, RUYES specifically created supplemental instruction in cancer biology for the student participants. This includes T*opics in Cancer Biology* sessions by senior graduate students and post-doctoral fellows at the Rutgers Cancer Institute, which include (not limited to) topics such as oncogenes, tumor suppressors, apoptosis, signaling pathways in cancer. Additional support is provided through seminars held at Rutgers Cancer Institute including the Cancer Center Grand Rounds, Distinguished Lecture series, and Predoc/Postdoc Trainee seminars. Seminar attendance is followed by a 30-minute discussion on the research topic and career path of the speaker. Special lectures and professional enrichment activities organized specifically for the participants in the program include monthly journal clubs, “survival skill” presentations and career counseling by the Program Director. Teachers and students do podium presentations at the culmination of their summer research training experience at the Annual Research Day.

### Post Summer Program Engagement.

We also believe that continuity of training experience rather than short summer experiences may be better for engaging participants for the long-term. Thus, RUYES teachers and students engage in 2–4 weeks of post-summer program activities to engage them throughout the academic year. Post-summer activities may include (not limited to), monthly check-in sessions for all participants; engagement in online Professional Learning Communities; outreach activities in first and second year of participation; preparation of posters/abstracts for presentation in local, regional and national meetings and testing and implementing curriculum developed during summer (only for teachers). RUYES program coordinators organize and track these activities.

### Outreach.

Several venues described below are used to conduct extensive outreach for recruitment, advertisement and to enhance the value of science education and research among families and communities of participants.

#### Program Website.

The RUYES provides information on various program components for teachers as well as students, faculty mentors and their areas of research, online application portal and eligibility and selection criteria. The program is cross-referenced on several other Rutgers websites including those of Pre-college Summer Programs, Rutgers Office of STEM Education and CMSCE.

#### Brochures and Flyers.

Paper and electronic versions of the brochures and flyers detailing program components including the eligibility and selection criteria, program benefits to participants, and contact information for program staff. These flyers are mailed to all the middle and high schools and science departments of colleges and universities across New Jersey, which has 731 middle schools, 422 high schools and 48 four-year colleges and universities.

#### Collaborations with Other Programs.

We work with the 4-H Youth Programs at Rutgers to distribute flyers and brochures introducing the program to all 21 counties across Rutgers. The 4-H program has monthly county fairs for youth involvement, which serve as an excellent venue for distributing print material for program recruitment. The Office of Community Outreach and Engagement and Office of Communications at Rutgers Cancer Institute also assists in recruitment efforts by distributing flyers and information about the program at community outreach events throughout the state. In addition, we advertise the program through the network of National Science Foundation Robert Noyce Teacher Scholarship Program and Science Education and Literacy Center (SELECT) for STEM teachers at Rider University.

To nurture supportive learning environments where science education and research are valued, we conduct outreach to middle school students, their families and other community members. The objective of the outreach efforts is to increase knowledge and awareness of cancer research, importance of general health and science education and biomedical, cancer research careers. Positive social contextual relationships such as parental support/encouragement positively affects an individual’s learning behavior and career choice (Ferry et al., 2000; Mallinckrodt, 2000). Studies show that in general, science achievement is influenced by parental involvement and support (Mallinckrodt, 2000; Scott and Mallinckrodt, 2005; Baker and Leary, 2003). We think that involving parents and communities of students, especially in the case of high school students is critical for student participation and retention in the program, since many of the high school students are still learning time management and juggling multiple commitments. Furthermore, social and cultural context has a strong influence on the career decision of minority youth (Chaudhary, 2015; Bhatt and Chaudhary, 2020. RUYES engages the parents early on in the process to explain the program and request their commitment and support. Parents are invited to the annual culminating summer research symposium for the RUYES participants.

Each cohort of RUYES students and teachers together develop a different outreach initiative in first and second year of participation. Working together on both these events described below, benefits program participants, by engaging them with each other in teams and building a community of learners.

#### Science on the Move-Focus on Cancer.

This initiative leverages the expertise and network of the Rutgers Office of STEM Education, Science Explorer program to develop cancer relevant modules for outreach to middle schools. To maximize impact in schools and local communities, participants develop an interactive module that includes group activities while using tailored lesson plans, visual aids and age-appropriate handouts for middle school students. Rutgers Science Explorer fellows, who are usually graduate students or post-docs within the STEM disciplines with an interest in teaching and/or science communication, deliver this module together with students and teachers in the RUYES program to middle school students in their communities. Examples of modules developed during summer 2021 include those on skin cancer prevention and effects of vaping on lungs.

#### School Outreach Event.

Each cohort of students and teachers develop this outreach event during second year of their participation. The Office of Community Outreach and Engagement (COE) at Rutgers Cancer Institute supports the participants in developing and implementing this event by providing both didactic and experiential content related to the science of population health, implementation science and community outreach interventions during the weekly meetings in summer. The culminating project related to this teaching objective is an outreach event held in participant’s schools in their communities (e.g. during back to school nights or after school clubs).

## EVALUATION

Ongoing evaluation and feedback of all components of the program, and the participants is crucial for providing a high-quality experience, satisfaction of the mentoring faculty, and successful continuation of the program. The evaluation is important to track fidelity and to look at implementation and outcomes. It also helps to understand which aspects of the program are successful and what we might be able to improve upon in subsequent years of the program. An experienced external (to the program) evaluator at Rutgers School of Management and Labor Relations conducts the evaluation. Rutgers IRB approved the evaluation study. Summer 2021 was first year of RUYES Program implementation. We describe below the measures, methodology and preliminary results from the evaluation.

### Measures.

We list below evaluation questions and outcome measures aligned with the program activities and specific aims.

### Methods.

We plan to conduct a prospective, longitudinal evaluation of individual beliefs/attitudes, social support, and intent to pursue a research career in students from URG and high school science teachers participating in RUYES program over a period of 5 years. Participants complete a pre (once) and post survey (same survey administered at two different time points) as described below. Survey instruments (Pre and Post) examine knowledge and interest in biomedical research and biomedical careers, based on the Likert-scale. The first survey tracks current interest and expectations about the program and the following surveys look at changes in interest and results of the program. Survey administration time points include:

- Pre-matriculation survey- within one week of matriculating in RUYES
- Post-matriculation survey 1- at the end of the year 1 of participation in the program
- Post-matriculation survey 2 - at the end of the year 2 of participation in the program

Surveys are administered electronically via Qualtrics at the time points listed above.

Survey questions are drawn from validated surveys (Biomedical Career Path survey, Pre and Post survey) used in published papers resulting from a prior study (Chaudhary, 2015). Scientific research competency in teachers is measured by responses to two questions: a) To what extent the RUYES training program contributed to increased skills in conducting cancer research; and b) To what extent the RUYES training program contributed to increased knowledge of research in the field of cancer. Self-efficacy beliefs in teachers is measured by a 7 item scale that includes 2 questions about science research self-efficacy, 4 questions about communicating science, and 1 question about self-efficacy beliefs to create novel cancer-focused curriculum. Self-efficacy beliefs in students is measured using a 26 items scale described previously (Chaudhary, 2015).

The evaluation methods also include interviews with study subjects. These interviews look at their experiences with the programs and their thoughts about what works and what could be improved. We also probe into their thoughts on how they will use what they have learned as they move forward in their classrooms or with their own educational experiences. Interviews are conducted by the external evaluator at 2 time points. Interview questions are based on attached interview guide used in a prior published research study (Bhatt and Chaudhary, 2020) and conducted at the following two time points:

- Post-matriculation interview 1- at the end of the year 1 of participation in the program
- Post-matriculation interview 2- at the end of the year 2 of participation in the program

### Preliminary Findings from Pilot Study.

We report below preliminary evaluation data collected from RUYES participants in summer 2021.

***Q1***. Is the RUYES program effective in reaching intended target populations of teachers and underrepresented students?

**Figure 1.**
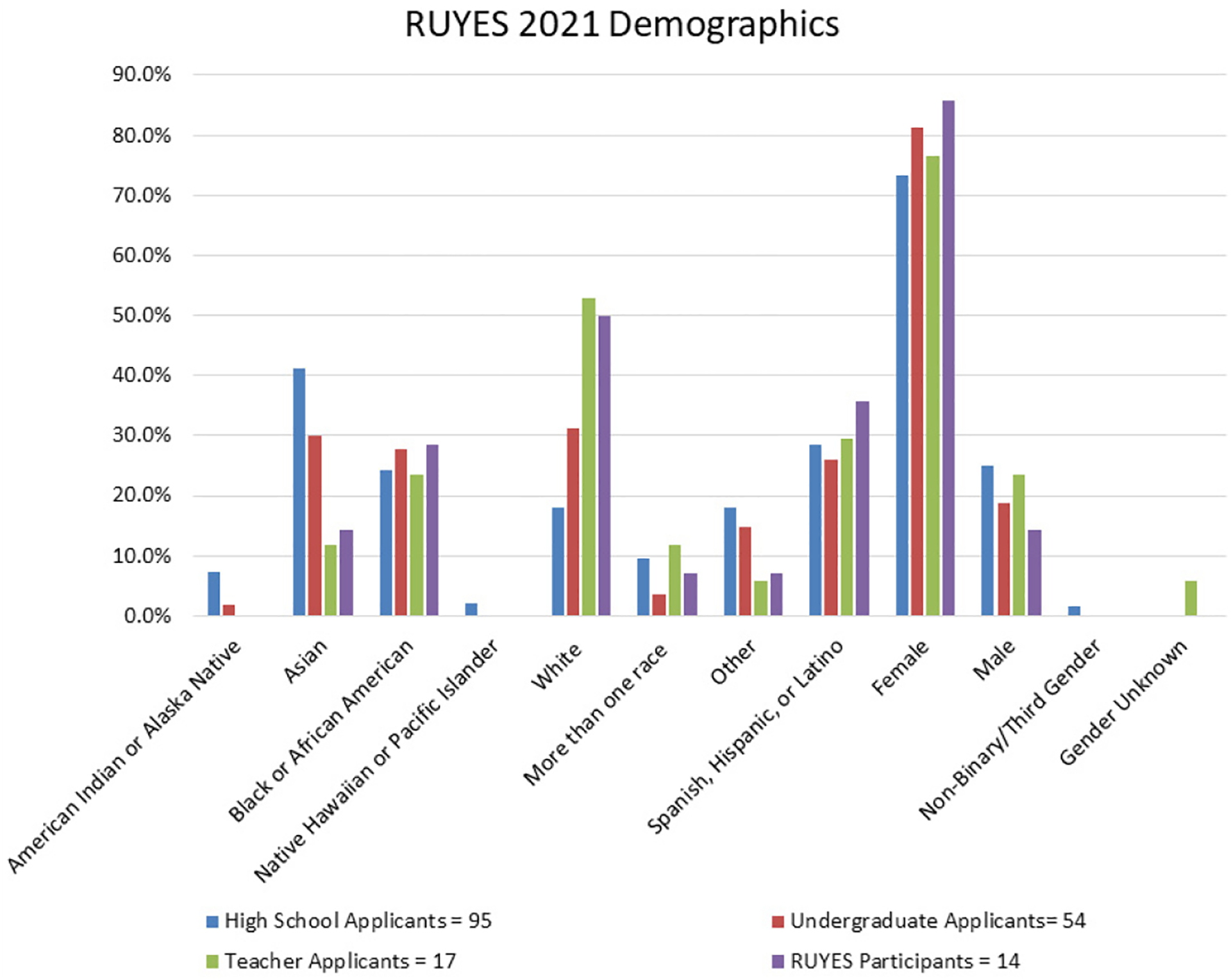
RUYES 2021 Demographics.

For both teacher and student applicants, RUYES recruitment efforts were concentrated in Camden, Essex, Hudson, Middlesex, and Passaic counties of the state of New Jersey as these areas have large urban centers and significant populations of underrepresented students. RUYES participants were selected from 166 applicants (17 applications from high school teachers, 54 undergraduate students, and 95 high school students). While teachers were not required to come from underrepresented backgrounds, a diverse applicant pool was sought during recruitment outreach (Figure 1). Racial and ethnic diversity increased among the 54 undergraduate full applicants (Figure 1). The high school student applicant pool included the largest percentage of racial and ethnic minorities (Figure 1). Applicants were encouraged to select all races/ethnicities that applied to them therefore, totals equal more than 100%. Females are underrepresented in the sciences and we were encouraged by the number of women who applied. Teacher applicants were overwhelmingly female at 76.5%, with male applicants at 23.5% and 5.9% of applicants not indicating their gender. Undergraduate students were 81.2% female and 18.8% male. Finally, high school students were 73.4% female, 25.0% male, and 1.6% were non-binary/third gender.

Fourteen individuals were selected to participate in the 2021 RUYES summer program- four high school teachers, five undergraduate students, and five high school students. Eleven of the fourteen participants come from school districts in the targeted counties (Essex=1, Hudson=3, Middlesex=7).

The Application Review Committee endeavored to select a diverse pool of program participants. Of the 14, seven are white; five are Spanish, Hispanic, or Latino; four are Black or African American; two are Asian; one is from more than one race; and one is from a race not listed here. As with the applicant pool, participants were encouraged to select all races and ethnicities that apply to them, and so numbers will total more than 100%. Twelve of last year’s participants were female and two were male (Figure 1).

***Q2***. To what extent does RUYES program provide effective mentoring, high quality hands-on research training, and professional development support?

The high school science teacher cohort was overall positive about their experiences with the program. Most teachers indicated that their expectations of the programs were met or exceeded. The high school student cohort was also positive about the program, and survey data reflected the program had a positive influence on their thinking about career choices and education. The undergraduate student group reported having a mixed experience with the program. Students indicated the pace and intensity of the program were challenging and felt the program was meant for students with more advanced skills than they possessed.

### Quotes from Participants

#### Successes

-
  “I love the lab experience. I have never had that opportunity, so now I know what our students need to succeed in college…I love that I have contacts with scientists that I can now bring into the classrooms.”
  Teacher 1
-
  “I honestly loved going to the lab for the first week. I even told my mentor how I loved coming there and how exciting it was.”
  Student 1
-
  “Prior to this program, I had never even considered research at all. It wasn’t something that I thought about. So, it has kind of opened a new door.”
  Student 2

#### Challenges

-
  “One thing I would change is that Fridays are jam-packed. The week is already hectic. From Monday-Thursday, I leave at 4:30–5:00, so that’s a whole day and there is not much down time.”
  Teacher 1
-
  “It gets jam packed between reading. If we can get assignments earlier, it would be easier to schedule work with them and distribute our time better.”
  Teacher 2
-
  “I feel like there was a little either miscommunication or just poor communication, specifically with my mentor, because I think she expected me to have a lot more knowledge coming into the lab than I had.”
  Student 1
-
  “It is a lot of time and a lot of work. But I definitely appreciate my research team. They were really accommodating if I didn’t have enough time to do something. I was able to accomplish a lot.”
  Student 2
-
  “The only dislike I have is that it is very time consuming, but I guess I have to realize that comes with more responsibility and entering the workforce.”
  Student 3

***Q3***. Did participation in the RUYES program contribute to advancing self-reported scientific research competencies in teachers, and science research self-efficacy beliefs in teachers and students?

##### Teachers.

Three of the four teachers reported high impact and one teacher reported moderate impact of the contribution of RUYES on their scientific research competency. No significant changes in science research self-efficacy and science communication skills of teachers were observed because of participation in RUYES. Three of the four teachers reported high contribution of RUYES on creating NGSS aligned novel cancer focused curriculum. During further probing during focus groups teachers reported the program contributed to advancing their competencies in relevant areas of scientific research: “*Being back in the lab is the best thing. I am learning new lab techniques and I can’t say enough about that*” (**Teacher 1**).

##### Students.

The high school student cohort reported an increase in confidence about science research skills from preto post- surveys. The only areas where some students remained less confident were in obtaining laboratory data and performing experimental procedures. This may be because not all students participated in lab-based projects.

The undergraduate student group reported having a mixed experience with the program. Student self-efficacy beliefs in their research skills and magnitude of interest in scientific research activities declined slightly in some areas from preto post-survey results.

Five students reported high and four students reported moderate contribution of RUYES on their confidence to “*Understand how scientific research is carried out*.” Other items on the 26 item self-efficacy scale that were rated high by students included, “*work with other collaboratively on a scientific project*” and “*think critically about scientific findings you read in the media*.” During focus group interviews, students indicated program’s contribution to increased confidence:

I think I got a lot more confident. In school, we’ve maybe done some things in science classes that are slightly reminiscent of the things some of us did during this summer, but I don’t think anything really compares to how in-depth and complex we were involved. I don’t think I have been ever able to immerse myself that much, so it’s definitely given me a lot more confidence.

(**Student** 1)

***Q4***. To what extent do students increase their knowledge of career options in biomedical, cancer workforce and intent to pursue science research careers?

Four out of the five undergraduate student’s response to survey questions indicated that the program influenced their career interests, with three of the four stating they wanted to enter research or cancer research careers, and the fourth stating they hoped to pursue a medical degree. All five undergraduate students plan on registering for a science course next semester.

All high school students indicated that the program had a positive influence on their career and science course-taking decisions. They all reported they will take science courses in the fall semester, including some Advanced Placement courses. After completion of the program, high school students expressed increased interest in pursuing important educational elements that can lead to a scientific career (i.e., reading scientific articles, taking science courses, majoring in a scientific field). Notably, students reported an increased interest in reading articles about science. These responses were further voiced during focus group interviews.

Prior to this program…I kind of just had a general interest and idea that I want to do something related to science but not sure what specifically, so it has really helped me narrow it down and helped me make some decisions. Research is definitely something that I might consider now.

(Student 1)

***Q5***. Does participation in RUYES influence teacher’s interest in developing cancer-focused curriculum to take back to their classrooms?

In response to survey question, two teachers indicated they planned curricula changes based on participation in the program. One respondent said: “*I hope to start my unit on cells based on cancer research. I will be giving my students a PBL* [project-based lesson] *on cancer*.”

During interviews the teachers indicated that the “*The best thing was learning how to write curriculum and be a part of the scientific community*.”

## LESSONS LEARNED

Preliminary evaluation of the RUYES program indicates that it is successful in providing an engaging research experience and professional development support to students as well as teachers and increases science research self-efficacy beliefs and STEM/science research career motivation and intent. The undergraduate students reported a slight decrease in self-efficacy pre and post Program participation. One explanation for this could be that first-year undergraduates are overconfident about their self-efficacy beliefs (Talsma, 2019, 2020). Participation in the summer program provided a realistic experience of what the students did not know prior to starting the Program and led to decreased science efficacy beliefs. An alternative explanation could be that this is a result of negative impacts of COVID-19 pandemic on the learning experience of students. This negative experience could be due to a confluence of several factors including lack of academic foundation and self-regulation skills; decreased social interactions, and heightened anxiety for the students (Talsma, 2021). Similar stressors may have also been present for the peer-mentors guiding the students in their research projects. To address this issue, the RUYES program will provide specific mentor skills training through National Research Mentoring Network to all peer-mentors. In addition, the RUYES program is in-person in summer 2022, allowing more opportunities for social interactions and dialogues with trainees to address academic anxiety issues. Conducting a skill-specific intake survey or interview with incoming students could also help ensure that program staff and mentors clearly understand the skills students are entering with, design specific training modules that students need to be successful, and effectively communicate participant skill level to mentors.

Participants, in terms of both deadlines and the general requirements of the program, reported time commitment as the biggest challenge. Pandemic related adjustments to the program made it necessary to have professional development activities, virtually scheduled on one day (Friday). Many participants in both the teacher and undergraduate student cohorts specifically identified Fridays as consistently being the most stressful day of the week due to the length of time they were required to be online. Going forward, we have spread out the curriculum support instructions, professional development and outreach activities in short 1–2 hour blocks spread across 3 days of the week.

Research training programs such as YES benefit not only the student participants but also the institution that supports them. Through such programs, the institution provides critical outreach function to the local community; forms networking alliances with other regional and national programs; brings in additional funding to support similar programs; ensures a candidate pool of future graduate and post-graduate students and gains fresh perspective and intellectual inquiry in the laboratories. Tangible benefits of participation for the students include an opportunity to gain valuable research experience, networking and mentorship from experienced scientists and clinicians, recommendation letters for admission into medical school, graduate programs etc., financial support, opportunity to do research thesis credits etc. Thoughtful Process and Outcome evaluation of research training programs is critical for Program participants, administrators, funding agencies, policy makers and other stakeholders.

## Figures and Tables

**Table 1.** Research Questions, Measures and Instrument.

Questions	Metrics	Instrument
**Process Evaluation**		
**Q1**. Is the RUYES program effective in reaching intended target populations of teachers and underrepresented students?	Number of applicants and participating trainees Demographics (race, gender, SES and disability status) of all applicants and trainees	Demographic Questionnaire
**Q2**. To what extent does RUYES program provide effective mentoring, high quality hands-on research training, and professional development support?	Satisfaction (attitudes/beliefs) with program components e.g. research mentors, research experience, laboratory skills training, sense of belongingness and professional development activities	Survey Questions: *Ratings of Program contribution to knowledge of research in the field of cancer and skills in conducting cancer research* *To what extent does the program meet trainee expectations* *Overall ratings of research experience* Focus group Interviews
**Outcome Evaluation**		
**Q3**. Did participation in the RUYES program contribute to increase in self-reported scientific research competencies, and science research self-efficacy beliefs in teachers and students?	Perceptions of increase in knowledge of and skills for cancer research Self-efficacy beliefs in science research	Survey Questions: TEACHERS *To what extent the RUYES training program contributed to increased skills in conducting cancer research; and* *To what extent the RUYES training program contributed to increased knowledge of research in the field of cancer*. STUDENTS Previously described 26-item science research self-efficacy scale to measure the changes in self-efficacy of participants of RUYES (Chaudhary, 2015).
**Q4**. To what extent do students increase their knowledge of career options in biomedical, cancer workforce and intent to pursue STEM careers?	What is the level of awareness, interest and motivation of students to pursue research careers after participation in RUYES?	Survey Questions: Has this research experience influenced your choice of subsequent science courses in any way? Do you intend to take the SAT, GRE, MCAT, apply to Graduate School? Has this research experience influenced your future career interests in any way?
**Q5**. Does participation in RUYES influence teacher’s interest in developing cancer-focused curriculum to take back to their classrooms?	What is the level of interest of teachers to develop cancer focused curriculum?	Survey Question: To what extent has RUYES contributed to increased interest in developing cancer-focused curriculum for high school students?

## ABBREVIATIONS

ASCO: American Society of Clinical Oncology
CIPT: Clinical Investigations and Precision Therapeutics
CMI: Cancer Metabolism and Immunology
CMSCE: Center for Mathematics, Science and Computer Education
CURE: Continuing Umbrella for Research Experience
GIGC: Genomic Instability and Cancer Genetics
LIF: Leukemia Inhibitory Factor
NBHSTHS: New Brunswick Health Sciences Technology High School
OCE: Office of Community Outreach and Engagement
PBL: Project-Based Lesson
PDAC: Pancreatic Ductal Adenocarcinoma
RUYES: Rutgers Youth Enjoy Science Program
SCCT: Social Cognitive Career Theory
SELECT: Science Education and Literacy Center
SPH: School of Public Health
TPB: Theory of Planned Behavior
URG: Underrepresented Backgrounds
